# Effectiveness of risk minimisation measures for valproate: A drug utilisation study in Europe

**DOI:** 10.1002/pds.5166

**Published:** 2020-11-23

**Authors:** Massoud Toussi, Margarita Shlaen, Florence Coste, Hanka de Voogd, Vasilis Dimos, Sigal Kaplan

**Affiliations:** ^1^ Department Real World Solutions IQVIA La Défense Cedex France; ^2^ Department Real World Solutions IQVIA Munich Germany; ^3^ Department Epidemiology and Risk‐Benefit Sanofi Aventis R&D Chilly Mazarin France; ^4^ Department Global Clinical Research Mylan Lyon France; ^5^ Department Pharmacovigilance (medical section) Demo S.A. Athens Greece; ^6^ Department Pharmacoepidemiology Teva Pharmaceutical Industries Ltd Netanya Israel

**Keywords:** drug utilisation study, risk minimisation measures, pharmacoepidemiology, valproate

## Abstract

**Purpose:**

The purpose of this study was to evaluate the effectiveness of the risk minimisation measures (RMMs) implemented in Europe in 2014 for valproate‐containing products to mitigate their risk during pregnancy and to characterise valproate prescribing patterns in women of childbearing potential (WCBP) before and after implementation of RMMs.

**Methods:**

A multinational cohort study based on existing data sources using a pre‐/post‐ design was performed in five European countries (France, Germany, Spain, Sweden, UK) in an outpatient setting. Effectiveness of RMMs was assessed by comparing the proportion of valproate initiations as second (or subsequent) line therapy before and after implementation of RMMs (primary outcome) with an increase in this proportion indicating success of RMMs. Overall use of valproate and incidence of pregnancies in WCBP were also examined.

**Results:**

The proportion of valproate initiations as second line therapy increased after implementation of RMMs in incident female users in Sweden (from 81.1%, 95% CI 79.9%‐82.3% to 84.5%, 95% CI 83.5%‐85.5%) and the UK (from 66.4%, 95% CI 64.5%‐68.3% to 72.4%, 95% CI 70.0%‐74.9%), it remained the same in Germany and Spain and decreased in France from 48.7% (95% CI 45.6%‐51.9%) to 40.6% (95% CI 37.6%‐43.7%). In Sweden and the UK, the incidence of pregnancies exposed to valproate decreased in the post‐implementation period: 8.0 vs 9.5 and 10.9 vs 16.9 per 1000 person‐years, respectively.

**Conclusion:**

The results on primary outcome of this study suggest limited effectiveness of the RMMs. Additional RMMs were implemented in 2018.

**KEY POINTS**

- This study provides insights to valproate prescribing patterns in five EU countries (France, Germany, Spain, Sweden and the UK) in the period before and after implementation of risk minimisation measures (RMMs) in 2014.
- The change in the proportion of valproate initiations as second (or subsequent) line therapy varied across countries and suggests limited effectiveness of the RMMs on this specific measure.
- The number of valproate prescriptions in all female patients and specifically in women of childbearing potential decreased after implementation of RMMs.
- A decrease in the incidence rates of pregnancies exposed to valproate suggests a positive effect of RMMs.

## 1 INTRODUCTION

Valproate containing medicines have been authorised for several decades across the European Union (EU) to treat epilepsy and bipolar disorder. In a few countries marketing authorisations are granted for prevention of migraine attacks. Numerous publications provide evidence on increased risks associated with valproate treatment during pregnancy. Approximately 10% of children exposed to valproate in the womb are at risk of congenital malformations. Developmental delays were reported for up to 30%‐40% of those children in pre‐school age.^1^, ^2^, ^3^, ^4^, ^5^, ^6^, ^7^ Furthermore, in utero exposure to valproate is associated with increased risk for autism spectrum disorder or symptoms of attention deficit/hyperactivity disorder (ADHD).^8^, ^9^, ^10^

Valproate medicines and their use in pregnant women was assessed by the European Medicines Agency (EMA) and triggered by a referral under Article 31 of Directive 2001/83/EC.^11^

Following regulatory restrictions recommended by the Pharmacovigilance Risk Assessment Committee (PRAC) in October 2014, several risk minimisation measures (RMMs) were implemented to mitigate the risk of valproate‐containing products in pregnancy.^12^ In accordance with the RMMs, “Valproate should not be used to treat epilepsy or bipolar disorder in girls and in women who are pregnant or who can become pregnant unless other treatments are ineffective or not tolerated. Women for whom valproate is the only option after trying other treatments, should use effective contraception and treatment should be started and supervised by a doctor experienced in treating these conditions. Women who have been prescribed valproate should not stop taking their medicine without first consulting their doctor. In countries where valproate medicines are authorised for the prevention of migraine, women must not use valproate for preventing migraine when they are pregnant. Pregnancy should be excluded before starting treatment for migraine, and women should use effective contraception. The PRAC also recommended that doctors who prescribe valproate provide women with full information to ensure understanding of the risks and to support their decisions.”^12^

The product information was amended accordingly. In addition, to improve awareness about the risks of valproate exposure during pregnancy, communication to healthcare professionals through a Direct Healthcare Professional Communication (DHPC) and educational materials (EMs) were provided to the healthcare professionals in the EU and to women prescribed valproate.^11^ The EM included a prescriber guide, a patient booklet, an acknowledgment of risk information form including a checklist for prescribers and a checklist for patients or carers.

Within the context of RMMs and in compliance with the EMA Guideline on good pharmacovigilance practices (GVP) Module XVI,^13^ two post‐authorisation safety studies (PASS), a drug utilisation study (DUS) and a prescriber survey, were conducted to assess the effectiveness of these RMMs and to further characterise the prescribing patterns for valproate.^14^

Here we present the results of the DUS in which prescribing practices and effectiveness of these measures were assessed in an outpatient setting by comparing the proportion of valproate initiations as second line therapy in females in general and specifically in women of childbearing potential (WCBP) before and after the implementation of RMMs (ie, dissemination of EMs and DHPC). The study was performed by a consortium of marketing authorization holders (MAHs) of valproate in Europe.

## 2 METHODS

### 2.1 Study design

**FIGURE 1:**
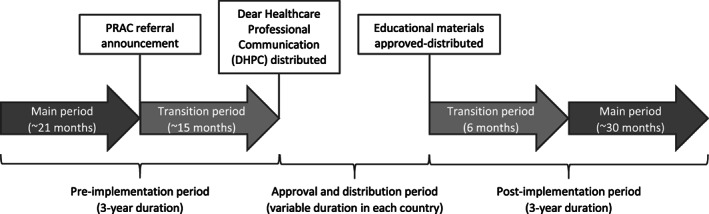
Study periods

This is a multinational non‐interventional cohort study performed in the outpatient setting in five European countries (France, Germany, Spain, Sweden and United Kingdom) using existing data sources. The DUS used a common protocol which was approved by PRAC and registered in the European EU PAS register (EUPAS9678). Ethic and data access approvals were obtained according to local regulations. Study countries were selected based on the availability of longitudinal databases and the high number of patients exposed to valproate. A pre‐post design was employed to examine the changes in prescribing of valproate after implementation of RMMs. Each three‐year pre‐ and post‐implementation period was divided into a main and a transition sub‐period (Figure 1).

The main pre‐implementation period started in January 2012 and ended at the time of the regulatory referral announcement (October 2013).^15^ The pre‐implementation transition period started at the time of the regulatory referral announcement and ended at the time of DHPC distribution (December 2014). The RMMs implementation period started at the DHPC distribution and ended at the EMs distribution (January to July 2015).

The post‐implementation period started after the distribution of the approved EMs by the health authorities in each country. The post‐implementation transition period comprised the first 6 months while the main post‐implementation period was defined as the subsequent 30 months (29 months in Sweden) after the end of the transition period.

### 2.2 Data sources

Data for this study were obtained from the following longitudinal patient‐level data sources: electronic medical records (EMR) database IMS Disease Analyzer (DA) for France and Germany, EMR IMS Longitudinal Patient Data (LPD) database for Spain, EMR Clinical Practice Research Datalink (CPRD) GOLD database including CPRD Pregnancy Register for the United Kingdom (UK), and three national health registers (Patient Register; Drug Register, Medical Birth Register) for Sweden. These databases contain information on patient's demographics, diagnoses and drug treatment. In the EMR databases, data are derived from a physician sample by specialty. In Sweden, all specialties are presented in the national health registers. An overview of the databases is provided in Table 1.

**TABLE 1:** An overview of the databases and parameters availability

	France	Germany	Spain	Sweden	UK
Data source	Disease Analyzer (DA)	Disease Analyzer (DA)	Longitudinal Patient Data (LPD)	National health registers: Patient Register; Drug Register, Medical Birth Register	Clinical Practice Research Datalink (CPRD)^e^, incl. CPRD Pregnancy Register
Database type	Primary health care electronic medical record database	Primary health care electronic medical record database	Primary health care electronic medical record database	National health record databases linked through the unique civil personal registration number	Primary health care electronic medical record database
Region captured	Country (Metropolitan France)	Country	Three regions of Spain^a^	Country	Country
Physician specialties captured in data source	GPs	GPs, specialists (eg, neurologists, psychiatrists, cardiologists, rheumatologists, gastroenterologists, gynaecologists)	GPs, specialists (eg, neurologists, psychiatrists, cardiologists, rheumatologists, gastroenterologists, gynaecologists)	All specialties	GPs
Coverage	~1.5 million patients (~2% of general population in France)^b^	~5 million patients (~6% of general population in Germany)^c^	~1 million patients (~2% of general population in Spain)^d^	Nationwide	~5 million patients (~8% of the UK population)^16^
Patients insurance status	No restrictions	No restrictions	No restrictions	No restrictions	No restrictions
Parameter captured:					
Demographics	Yes	Yes	Yes	Yes	Yes
Medications	Yes (prescriptions)	Yes (prescriptions)	Yes (prescriptions)	Yes (pharmacy‐dispensed prescriptions)	Yes (prescriptions)
Diagnoses	Yes	Yes	Yes	Yes	Yes
Pregnancy	Restricted	Restricted	Restricted	Yes	Yes^f^

Abbreviation: GP, general practitioner.

^a^ Regions cannot be listed for LPD data protection reasons.

^b^ Data from 2018.

^c^ Data from 2017‐2019, GPs.

^d^ Data from 2019.

^e^ CPRD GOLD.

^f^ CPRD Pregnancy register.

The CPRD (UK) and DA France provide a nationally representative sample of primary care (general practitioners, GPs). In Germany and Spain, data from the primary care physicians (PCP) panel and the neurologists/psychiatrists panel were used to account for the majority of valproate prescribers. The PCP panel includes GPs and internists in Germany and GPs and paediatricians in Spain.

### 2.3 Population

The study population included all female patients receiving at least one prescription of an oral formulation of valproate in the outpatient setting during the study period in the selected databases. The main analysis unit was the prescription. Prescriptions for women aged 13 to 49 years at the prescription date were included in the WCBP subgroup.

Valproate prescriptions issued during the study period were defined as „incident“ if there was no use of valproate within 12 months prior to the prescription date; "first‐ever“ valproate prescriptions were defined as no use of valproate documented during the patient's entire medical history. To avoid misclassification due to short medical history, classification as „incident“ or "first‐ever“ prescription required medical history available in the database for at least 12 months prior to the valproate prescription date.

### 2.4 Outcome parameters

#### 2.4.1 Primary outcome

Given the safety concern that led to the prescription condition “Valproate should not be used to treat epilepsy or bipolar disorder in girls and in women who are pregnant or who can become pregnant unless other treatments are ineffective or not tolerated” the study aimed to assess valproate use as second (or subsequent) line therapy. The proportion of valproate initiations preceded by at least one other medication for valproate indications (epilepsy or bipolar disorder or migraine) was used as the operational definition for a second (or subsequent) line therapy (list of other medications is provided in the Appendix S5 in Data S1). Change in this proportion after the implementation of RMMs was defined as primary outcome of this study. An increase in this proportion in the main post‐implementation period compared to the main pre‐implementation period was defined as success of RMMs.

Data on prior medication was obtained from the 12‐month period prior to valproate initiation in the analysis for the incident valproate prescriptions and from patients' entire medical history for the first‐ever prescriptions. Analysis of the primary outcome parameter was performed for all valproate initiations (incident and first‐ever prescriptions), for the subgroup of WCBP and additionally for the subgroups of epilepsy and bipolar disorder indications. These subgroups were defined as valproate prescriptions issued for treatment of epilepsy or bipolar disorder. A detailed description of the algorithm used for the analysis of indication is available in Appendix S4 in Data S1.

#### 2.4.2 Pregnancy

Changes in the proportion of pregnancies were analysed in WCBP for the entire 36‐month pre‐ and post‐implementation periods. Of note, for Sweden data was available for only 23 months of the post‐implementation period for this analysis. Pregnancies were identified via ICD‐10 codes. The overall number of pregnancies as well as the number of pregnancies exposed to valproate were reported. A pregnancy was considered as “exposed” if at least one valproate prescription was recorded during pregnancy. In Sweden, pregnancy start and end date were mainly derived from last menstrual period (LMP) or delivery date recorded in the Medical Birth register. In the CPRD Pregnancy Register in UK, estimated start and end date of pregnancy were available based on an algorithm described elsewhere.^17^, ^18^ In the physician panels used for France, Germany and Spain, pregnancies could mainly be identified in case of medical visits due to pregnancy complications or adverse events; information on start/end date was usually not available. If information on the pregnancy start/end date was not available in the data source, the pregnancy was considered as “exposed” if at least one valproate prescription was issued between 90 days before and 180 days after the first record related to pregnancy.

### 2.5 Statistical analysis

Data were analysed at the prescription level by country and, in Germany and Spain, by physician panel (PCP and neurologists/psychiatrists) separately. Number of valproate prescriptions per study period was reported. Age was assessed at the date of each prescription and patient age was defined as age at the first prescription during the study period. Changes in prescribing behaviour before and after implementation of RMMs were primarily assessed using data from the two main study periods. The main study periods were used to ascertain the effectiveness of RMMs regardless of transition periods around their implementation. The entire 36‐month pre‐ and post‐implementation periods were used to analyse the incidence of pregnancies.

All analyses were performed by descriptive statistical methods. The results of the primary outcome were calculated as proportions (%) with 95% confidence intervals (CI). Non‐overlapping 95% CIs indicated a significant difference between pre‐ and post‐implementation periods. The incidence of pregnancies in WCBP was estimated per 1000 person‐years. Data were analysed using SAS System 9.3 or higher (SAS Inc., Cary/NC, US).

## 3 RESULTS

The number of women included in the study ranged from 1683 (1501 in the PCP and 182 in the neurologists/psychiatrists panels) in Spain to 14 287 in Sweden in the main pre‐implementation period and from 1839 (1686 in the PCP and 153 in the neurologists/psychiatrists panel) in Spain to 14 444 in Sweden in the main post‐implementation period (Table 2). The overall number of valproate prescriptions varied between 11 052 in France and 184 606 in Sweden in the main pre‐ and between 12 555 in France and 257 573 in Sweden in the main post‐implementation period. Information about incident and first‐ever valproate prescriptions is summarised in Table 2. Proportion of prescriptions that could not be classified as incident or first ever prescriptions due to short medical history varied between 0% in Sweden, under 1% in Spain, around 5% in Germany, 9% in France and up to 12% in the UK.

**TABLE 2:** Number patients and valproate prescriptions in the main study periods

	Overall		WCBP (Age group 13 to 49 y)	
	Main pre‐implementation period	Main post‐implementation period^a^	Main pre‐implementation period	Main post‐implementation period^a^
**Patients**				
***France***	3013	2645	1444	977
***Germany***				
PCP panel	2748	2792	944	844
Neurologists/Psychiatrist panel	4743	4141	2061	1618
***Spain***				
PCP panel	1501	1686	712	709
Neurologists/Psychiatrist panel	182	153	111	80
***Sweden*** ^***1***^	14 287	14 444	6865	6661
***UK***	12 356	7952	5098	2950
**Prescriptions (overall)**				
***France***	11 052	12 555	4460	3572
***Germany***				
PCP panel	12 953	15 048	4438	4454
Neurologists/Psychiatrist panel	23 484	25 567	9693	8874
***Spain***				
PCP panel	13 663	20 742	6193	8305
Neurologists/Psychiatrist panel	740	519	393	243
***Sweden*** ^***1***^	184 606	257 573	74 224	96 397
***UK***	178 938	142 995	64 315	46 509
**Incident prescriptions**				
***France***	969	1002	521	403
***Germany***				
PCP panel	947	1214	286	350
Neurologists/Psychiatrist panel	1171	1260	512	499
***Spain***				
PCP panel	449	554	206	237
Neurologists/Psychiatrist panel	127	146	77	79
***Sweden***	4424	5065	2417	2659
***UK***	2367	1269	945	530
**First‐ever prescriptions**				
***France***	542	465	289	165
***Germany***				
PCP panel	691	827	188	202
Neurologists/Psychiatrist panel	666	679	275	241
***Spain***				
PCP panel	363	337	157	133
Neurologists/Psychiatrist panel	116	125	71	66
***Sweden***	3676	3972	1982	2008
***UK***	2007	1026	735	403

^a^ Duration of the main pre‐implementation period 21 mo, post‐implementation period – 30 mo (in Sweden: 29 mo).

Comparing the entire 36 months pre‐ and post‐implementation periods, the overall number of valproate prescriptions decreased between 2.7% and 37.8% after implementation of RMMs in all countries with the exception of Spain (Table 3). The decrease was even more pronounced in the WCBP subgroup than in the overall population. The number of incident and first‐ever valproate prescriptions also decreased– in most countries to a greater extent in the WCBP subgroup than in the overall population.

**TABLE 3:** Number of valproate prescriptions in the entire pre‐ and post‐implementation study periods

	Overall		WCBP (Age group 13 to 49 y)	
	Entire pre‐implementation period	Entire post‐implementation period^a^	Entire pre‐implementation period	Entire post‐implementation period^a^
	N	N (% change^b^)	N	N (% change^b^)
**All valproate prescriptions**				
***France***	19 634	15 790 (−19.6)	7686	4720 (−38.6)
***Germany***				
PCP panel	22 137	18 639 (−15.8)	7456	5593 (−25.0)
Neurologists/Psychiatrist panel	39 861	31 974 (−19.8)	16 168	11 266 (−30.3)
***Spain***				
PCP panel	23 844	25 014 (+4.9)	10 624	10 092 (−5.0)
Neurologists/Psychiatrist panel	1414	786 (−44.4)	767	372 (−51.5)
***Sweden***	319 524	310 889 (−2.7)	127 499	117 063 (−8.2)
***UK***	288 497	179 536 (−37.8)	101 806	58 996 (−42.1)
**Incident valproate prescriptions**				
***France***	1656	1238 (−25.2)	849	513 (−39.6)
***Germany***				
PCP panel	1629	1468 (−9.9)	492	416 (−15.4)
Neurologists/Psychiatrist panel	1978	1577 (−20.3)	835	632 (−24.3)
***Spain***				
PCP panel	758	652 (−14.0)	353	286 (−19.0)
Neurologists/Psychiatrist panel	247	177 (−28.3)	152	99 (−34.9)
***Sweden***	7521	6139 (−18.4)	4122	3252 (−21.1)
***UK***	3748	1667 (−55.5)	1506	687 (−54.4)
**First‐ever valproate prescriptions**				
***France***	925	573 (−38.1)	475	212 (−55.4)
***Germany***				
PCP panel	1168	995 (−14.8)	309	236 (−23.6)
Neurologists/ psychiatrist panel	1147	867 (−24.4)	456	316 (−30.7)
***Spain***				
PCP panel	577	402 (−30.3)	252	165 (−34.5)
Neurologists/ psychiatrist panel	220	153 (−30.5)	133	85 (−36.1)
***Sweden***	6180	4827 (−21.9)	3327	2477 (−25.5)
***UK***	3169	1354 (−57.3)	1186	524 (−55.8)

^a^ Duration of entire pre‐ and post‐implementation periods was 36 mo; in Sweden – entire post‐implementation period – 35 mo.

^b^ Change of prescription number in the entire post‐implementation period in comparison to the entire pre‐implementation period.

The mean age of patients receiving valproate in the main pre‐ and post‐implementation periods ranged from 45.3 (SD = 21.8) years in Sweden to 58.5 (SD = 21.3) years in Germany. WCBP who were prescribed valproate accounted for 34.5% (n = 943, PCP panel in Germany) to 61.0% (n = 111, neurologists/psychiatrists panel in Spain) of the overall population in the main pre‐implementation period (Table 4). This proportion decreased in the main post‐implementation period in all countries and ranged from 30.1% (n = 839) in the German neurologists/psychiatrists panel to 52.3% (n = 80) in the neurologists/psychiatrists panel in Spain.

**TABLE 4:** Patients age at start of main study periods: overall study population

	Main study periods	N patients total	N patients with non‐missing values^a^	Age group^b^		
				0‐12 y n (%)	13‐49 y n (%)	≥50 y n (%)
**France**	Pre‐implementation	3013	3005 (99.7)	148 (4.9)	1444 (48.1)	1413 (47.0)
	Post‐implementation	2645	2644 (100.0)	103 (3.8)	977 (37.0)	1564 (59.2)
**Germany PCP**	Pre‐implementation	2748	2737 (99.6)	41 (1.5)	943 (34.5)	1753 (64.0)
	Post‐implementation	2792	2789 (99.9)	54 (1.9)	839 (30.1)	1896 (68.0)
**Germany Neurologists/Psychiatrists**	Pre‐implementation	4743	4741 (100.0)	7(0.2)	2058 (43.4)	2676 (56.4)
	Post‐implementation	4141	4138 (99.9)	8(0.2)	1618 (39.1)	2512 (60.7)
**Spain PCP**	Pre‐implementation	1501	1492 (99.4)	117 (7.8)	701 (47.0)	674 (45.2)
	Post‐implementation	1686	1681 (99.7)	97 (5.8)	702 (41.8)	882 (52.4)
**Spain Neurologists/Psychiatrists**	Pre‐implementation	182	182 (100.0)	0 (0.0)	111 (61.0)	71 (39.0)
	Post‐implementation	153	153 (100.0)	0 (0.0)	80 (52.3)	73 (47.7)
**Sweden**	Pre‐implementation	14 287	14 287 (100.0)	1162 (8.1)	6865 (48.1)	6260 (43.8)
	Post‐implementation	14 444	14 444 (100.0)	1219 (8.5)	6661 (46.1)	6564 (45.4)
**UK**	Pre‐implementation	12 356	12 356 (100.0)	717 (5.8)	5044 (40.8)	6595 (53.4)
	Post‐implementation	7952	7952 (100.0)	453 (5.7)	2910 (36.6)	4598 (57.7)

^a^ Percentage of total N patients.

^b^ Percentage of N patients with non‐missing values.

### 3.1 Initiation of valproate as a second (or subsequent) line therapy (primary outcome)

**FIGURE 2:**
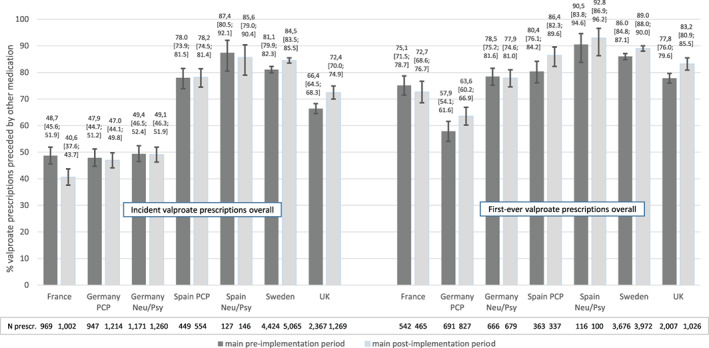
Initiation of valproate as second line therapy: proportion of valproate initiations preceded by other medications for valproate indications. Overall study population; main study periods; all incident and first‐ever prescriptions [Colour figure can be viewed at wileyonlinelibrary.com]

**FIGURE 3:**
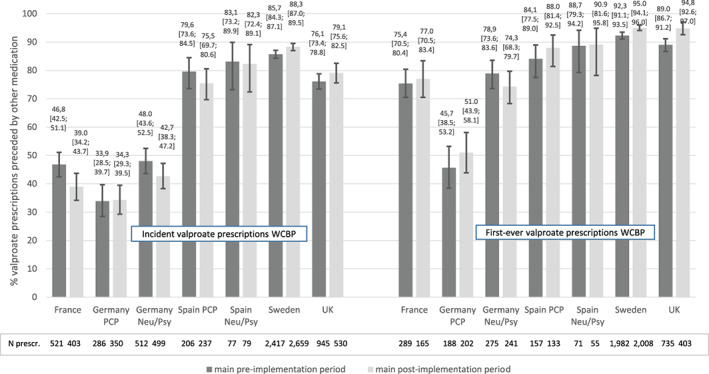
Initiation of valproate as second line therapy: proportion of valproate initiations preceded by other medications for valproate indications. Subgroup WCBP; main study periods; incident prescriptions and first‐ever prescriptions [Colour figure can be viewed at wileyonlinelibrary.com]

For incident prescriptions, the overall proportion of valproate initiations as second (or subsequent) line therapy was similar in the main pre‐ and post‐implementation periods in Germany (PCP panel: 47.9% vs 47.0%; neurologists/psychiatrists panel: 49.4% vs 49.1%) and in the PCP panel in Spain (78.0% vs 78.2%, see Figure 2 and Table S1 in Data S1). However, this proportion was higher in the main post‐ than in the main pre‐implementation period in the UK (72.4% vs 66.4%) and Sweden (84.5% vs 81.1%) and lower in France (40.6% vs 48.7%). These differences in France, Sweden and the UK were statistically significant as indicated by the non‐overlapping 95% CIs of the two periods. For incident prescriptions in the WCBP subgroup, the proportion of valproate initiations as second (or subsequent) line therapy increased in the main post‐implementation period by about 3% in Sweden and the UK and decreased by 7.8% in France and 5.3% in Germany (neurologists/psychiatrists panel, see Figure 3 and Table S1 in Data S1).

For first‐ever valproate users, the proportion of valproate initiations as second (or subsequent) line therapy was higher compared to incident valproate users in all countries in both periods. For WCBP, an increase in this proportion from the main pre‐ to post‐implementation period was observed in all countries with exception of the German neurologists/psychiatrists panel. In Sweden and the UK, this increase was statistically significant in all first‐ever valproate users and in WCBP.

In the indication subgroup “epilepsy” the proportion of incident prescriptions preceded by an indication specific medication (valproate as second line therapy) was higher in the main post‐ than in main pre‐implementation period in Sweden (56.3% vs 47.2%), the UK (44.1% vs 36.2%) and in the PCP panel in Spain (47.3% vs 41.3%); it was similar in both periods in Germany (PCP: about 21% in both periods; neurologists/psychiatrists panel: 26.4% in the pre‐ and 23.2% in the post‐implementation period, see Table S2 in Data S1). In France the proportion was lower in the post‐implementation period: 7.4% vs 19.7%. Under consideration of non‐overlapping 95% CIs, the differences between two periods were statistically significant in Sweden and France.

In the subgroup “bipolar disorder” the proportion of valproate initiations as second line therapy in incident prescriptions was slightly higher in the post‐implementation period in the UK (77.8% vs 72.9%) and was similar in both periods in Sweden (about 90%), Germany (PCP: about 38%; neurologists/psychiatrists panel: about 65%) and the PCP panel in Spain (about 90%). In France, the proportion was lower in the post‐implementation period (45.4% vs 54.8%, see Table S2 in Data S1).

The results for first‐ever valproate users were consistent with those for incident users in both “epilepsy” and “bipolar disorder” subgroups (Table S2 in Data S1). However, the proportion was generally higher for first‐ever users than for all incident users. Based on the non‐overlapping 95% CIs, the difference between the two main study periods was statistically significant in both indication subgroups in Sweden.

### 3.2 Pregnancy

In total, 451 of 923 pregnancies (48.9%) were exposed to valproate in the entire pre‐implementation period and 182 of 350 pregnancies (52.0%) in the entire post‐implementation period in all five target countries (Table S3 in Data S1).

The most comprehensive data on pregnancy was available in the data sources for Sweden and the UK. In Sweden, 179 of 402 pregnancies (44.5%) exposed to valproate were identified in the entire pre‐ and 77 of 140 (55.0%) in the entire post‐implementation period. The corresponding figures in the UK were 214 of 435 (49.2%) and 85 of 177 (48.0%) exposed pregnancies in entire pre‐ and post‐implementation periods, respectively.

The number of pregnancies identified in the EMR databases in France and Spain was low (3 to 51); only one pregnancy was identified in Germany in the entire pre‐implementation period.

The incidence rate of pregnancies (overall and exposed to valproate) in WCBP was lower in the entire post‐implementation period than in the entire pre‐implementation period in all analysed countries (Table 5).

**TABLE 5:** Incidence rate of pregnancies in overall and exposed to valproate (per 1000 person‐years) in WCBP (age group 13 to 49 y); entire pre‐ and post‐implementation periods

	Incidence per 1000 person‐years (n pregnancies)	
	Entire pre‐implementation period	Entire post‐implementation period^a^
**France**		
All pregnancies	3.8 (14)	0.7 (2)
Pregnancies exposed to valproate	2.7 (10)	0.4 (1)
**Germany PCP**		
All pregnancies	n.a.	n.a.
Pregnancies exposed to valproate	n.a.	n.a.
**Germany Neurologists/Psychiatrists**		
All pregnancies	n.a.	n.a.
Pregnancies exposed to valproate	n.a.	n.a.
**Spain PCP, Neurologists/Psychiatrists**		
All pregnancies	23.4 (50)	14.9 (28)
Pregnancies exposed to valproate	17.4 (37)	8.5 (16)
**Sweden**		
All pregnancies	21.2 (401)	14.5 (140)
Pregnancies exposed to valproate	9.5 (179)	8.0 (77)
**UK**		
All pregnancies	34.3 (435)	22.7 (177)
Pregnancies exposed to valproate	16.9 (214)	10.9 (85)

Abbreviation: n.a., not available.

^a^ Duration of the entire post‐implementation period considered for analysis of pregnancy in Sweden was 23 mo.

## 4 DISCUSSION

This DUS provides insights in the prescribing practices of valproate before and after implementation of RMMs and the effectiveness of these RMMs in the outpatient setting in five European countries.

The change in the proportion of initial valproate prescriptions as second (or subsequent) line therapy from pre‐ to post‐ implementation period suggests limited effectiveness of the RMMs on this specific measure. It varied across countries. In Sweden and the UK, it showed an increase from pre‐ to post‐implementation period implying more appropriate second line prescribing. However, in France it decreased and in Germany and Spain it did not change. The low impact of RMMs in France has already been reported previously.^19^ The generally higher level of prior medication observed in Sweden and the UK even before the introduction of RMMs is at least partially due to the national coverage of registry data in Sweden and the gatekeeper role of GPs in the management of patients in the UK.

In both study periods and all countries, the proportion of valproate initiations for bipolar disorder preceded by other medications for this indication was considerably higher compared to epilepsy both in the overall study population and in WCBP. Patients with bipolar disorder are often exposed to polypharmacy due to the character of the disease with manic and depressive episodes. Valproate is indicated for treatment of the manic episodes in patients who cannot take lithium due to contraindication or intolerance. Therefore, it seems plausible that prior to valproate initiation, other drugs are more frequently administered in patients with bipolar disorder compared to epilepsy. For epilepsy patients, valproate seems to remain a common initial treatment option.

The overall number of valproate prescriptions in females decreased after the implementation of these RMMs in the majority of target countries, suggesting a change of prescribing behaviour and reduction of the total exposure to valproate. The largest reduction in valproate use was observed in the UK. This is consistent with previous studies.^20^, ^21^ An increase of practice deregistration in the CPRD GOLD in recent years could partially contribute to the reduction of the number of prescriptions recorded in the UK during the post‐implementation period, but it had no impact on the main conclusion of the study. A reduction of valproate initiation (incident as well as first‐ever use) in female patients after implementation of RMMs was observed in the overall patient population and even more pronounced in the WCBP subgroup in all target countries.

A meaningful decrease in valproate initiation in France and the UK was also suggested in a study by Charlton et al.^16^ For Sweden, this decrease was in line with a study by Karlsson Lind et al which found an overall significant decrease of valproate initiations 2 years after the implementation of RMMs in 2015.^22^

The most comprehensive data on pregnancy was available in the National Medical Birth Register in Sweden and in the UK, where the CPRD Pregnancy Register is a valid database for analysis of pregnancy.^18^ The observed decrease in incidence of pregnancies in Sweden and the UK might indicate a positive impact of the RMMs. Despite the decrease in pregnancy incidence, a number of pregnancies were still exposed to valproate during the post‐implementation period. This finding supported the relevance of the more recent additional RMMs, including pregnancy prevention programs, introduced in 2018, in order to further reduce the number of exposed pregnancies.

Our study has several limitations. In the EMR databases for France, Germany, and to some extent in Spain patients cannot be tracked across practices and specialties. Patients who seek care outside the EMR practice setting have no data recorded in the database. This may lead to misclassification of exposure among patients who initiate treatment by a specialist and receive follow‐up prescriptions by a GP (false incident prescriptions by GPs) as well as to underestimation of medication use.

Overestimation of the primary outcome may have been possible in the analysis of all valproate initiations, because medications for all valproate‐relevant diagnoses from the medical history were considered irrespective of the specific indication for valproate initiation. Nevertheless, even in case of overestimation, this applied to both study periods and therefore would not have impacted the comparison. In the analysis of indication subgroups this overestimation was not relevant because only indication‐specific medication was considered.

Information on pregnancy is not comprehensively captured in the EMR databases and specifically in the physician specialty panels selected for this study in France, Germany and Spain. In Sweden, not all pregnancies were captured because only pregnancies of ≥23 weeks gestational age are included in the Swedish Birth Register. In none of the countries, the underlying conditions for exposure to valproate during pregnancy could be elucidated, since information regarding the patient's knowledge of risks or the prescribing physician's decision could not be retrieved in this study.

A limitation of this study was the inclusion of all female patients, irrespective of their fertility status. As the proportion of infertile/sterile women is expected to be low in the population and is unlikely to vary between the pre‐ and post‐implementation periods, this limitation was considered minor with no impact on the results of comparative analyses.

On the other hand, this DUS has some major strengths. This study included extensive numbers of patients from large longitudinal databases in five European countries receiving valproate in the real‐world setting with a lengthy observation period of up to 6 years. No exclusion criteria potentially introducing selection bias or affecting the external validity of results were applied in the study.

Overall, this study indicates that the effectiveness of RMMs was limited with regard to the valproate initiations as a second line therapy. However, the decrease in the overall number of valproate initiations and incidence of pregnancies in Sweden and the UK (the countries with the most interpretable data on pregnancy) suggest a reduction of the total exposure and exposure in pregnancy.

In 2018, the EMA introduced additional RMMs to strengthen the previous restrictions on valproate use.^23^ Further studies are underway to evaluate effectiveness of these measures. An extension of this DUS is in preparation to further monitor the use of valproate in WCBP and particularly the occurrence of pregnancies exposed to valproate.

## ETHICS STATEMENT

All procedures performed in this study involving human participants were in accordance with the ethical standards of the applicable institutional review board and ethics committees and with the 1964 Declaration of Helsinki and its later amendments or comparable ethical standards. The protocol of the study was approved by the EMA and registered in the EU PAS register prior to the start of data collection.

## CONFLICT OF INTEREST

Massoud Toussi and Margarita Shlaen are salaried employees of IQVIA, a human data science company which received funds for the conduct of the study. Florence Coste is an employee of Sanofi Aventis R&D (France). Sigal Kaplan is an employee of Teva Pharmaceutical Industries Ltd. (Israel). Hanka de Voogd is an employee of Mylan EPD (France). Vasilis Dimos is an employee of DEMO S.A. (Greece).

## Supporting information


**Data S1.** Supporting information.
