# Supplementary material for: Effectiveness of risk minimisation measures for valproate: A drug utilisation study in Europe
Source: Pharmacoepidemiol Drug Saf. 2020 Nov 23;30(3):292–303. doi: 10.1002/pds.5166 (PMC7894134; doi:10.1002/pds.5166)
Supplement: Supplementary file 1 — Data S1. Supporting information. [file PDS-30-292-s001.docx]

**Effectiveness of Risk Minimisation Measures for Valproate: a Drug Utilisation Study in Europe**

# Supplementary Material

**Table S1** Initiation of valproate as second line therapy: proportion of valproate initiations preceded by other medications for valproate indications. Main study periods; incident prescriptions and first-ever prescriptions

|  | **Incident valproate prescriptions^†^** | | | | **First-ever valproate prescriptions^‡^** | | | |
| --- | --- | --- | --- | --- | --- | --- | --- | --- |
|  | **Overall** | | **WCBP (Age group 13 to 49 years)** | | **Overall** | | **WCBP (Age group 13 to 49 years)** | |
|  | **Main pre-implementation period n (%),  [95% CI of %]** | **Main post-implementation period n (%),  [95% CI of %]** | **Main pre-implementation period n (%),  [95% CI of %]** | **Main post-implementation period n (%),  [95% CI of %]** | **Main pre-implementation period n (%),  [95% CI of %]** | **Main post-implementation period n (%),  [95% CI of %]** | **Main pre-implementation period n (%),  [95% CI of %]** | **Main post-implementation period n (%),  [95% CI of %]** |
| **France** |  |  |  |  |  |  |  |  |
| ***N prescriptions*** | ***969*** | ***1,002*** | ***521*** | ***403*** | ***542*** | ***465*** | ***289*** | ***165*** |
| Prescriptions with prior medication for valproate indications | 472 (48.7) [45.6; 51.9] | 407 (40.6) [37.6; 43.7] | 244 (46.8) [42.5; 51.1] | 157 (39.0) [34.2; 43.7] | 407 (75.1) [71.5; 78.7] | 338 (72.7) [68.6; 76.7] | 218 (75.4) [70.5; 80.4] | 127 (77.0) [70.5; 83.4] |
| **Germany PCP** |  |  |  |  |  |  |  |  |
| ***N prescriptions*** | ***947*** | ***1,214*** | ***286*** | ***350*** | ***691*** | ***827*** | ***188*** | ***202*** |
| Prescriptions with prior medication for valproate indications | 454 (47.9) [44.7; 51.2] | 570 (47.0) [44.1; 49.8] | 97 (33.9) [28.5; 39.7] | 120 (34.3) [29.3; 39.5] | 400 (57.9) [54.1; 61.6] | 526 (63.6) [60.2; 66.9] | 86 (45.7) [38.5; 53.2] | 103 (51.0) [43.9; 58.1] |
| **Germany Neurologists/psychiatrists** |  |  |  |  |  |  |  |  |
| ***N prescriptions*** | ***1,171*** | **1,260** | ***512*** | ***499*** | ***666*** | ***679*** | ***275*** | ***241*** |
| Prescriptions with prior medication for valproate indications | 579 (49.4) [46.5; 52.4] | 619 (49.1) [46.3; 51.9] | 246 (48.0) [43.6; 52.5] | 213 (42.7) [38.3; 47.2] | 523 (78.5) [75.2; 81.6] | 529 (77.9) [74.6; 81.0] | 217 (78.9) [73.6; 83.6] | 179 (74.3) [68.3; 79.7] |
| **Spain PCP** |  |  |  |  |  |  |  |  |
| ***N prescriptions*** | ***449*** | ***554*** | ***206*** | ***237*** | ***363*** | ***337*** | ***157*** | ***133*** |
| Prescriptions with medication for valproate indications | 350 (78.0) [73.9; 81.5] | 433 (78.2) [74.5; 81.4] | 164 (79.6) [73.6; 84.5] | 179 (75.5) [69.7; 80.6] | 292 (80.4) [76.1; 84.2] | 291 (86.4) [82.3; 89.6] | 132 (84.1) [77.5; 89.0] | 117 (88.0) [81.4; 92.5] |
| **Spain Neurologists/psychiatrists** |  |  |  |  |  |  |  |  |
| ***N prescriptions*** | ***127*** | ***146*** | ***77*** | ***79*** | ***116*** | ***125*** | ***71*** | ***66*** |
| Prescriptions with medication related to valproate indications | 111 (87.4) [80.5; 92.1] | 125 (85.6) [79.0; 90.4] | 64 (83.1) [73.2; 89.9] | 65 (82.3) [72.4; 89.1] | 105 (90.5) [83.8; 94.6] | 116 (92.8) [86.9; 96.2] | 63 (88.7) [79.3; 94.2] | 60 (90.9) [81.6; 95.8] |
| **Sweden** |  |  |  |  |  |  |  |  |
| ***N prescriptions*** | ***4,424*** | ***5,065*** | ***2,417*** | ***2,659*** | ***3,676*** | ***3,972*** | ***1,982*** | ***2,008*** |
| Prescriptions with medication related to valproate indications | 3,588 (81.1) [79.9; 82.3] | 4,278 (84.5)  [83.5; 85.5] | 2,072 (85.7) [84.3; 87.1] | 2,347 (88.3) [87.0; 89.5] | 3,160 (86.0) [84.8; 87.1] | 3,535 (89.0) [88.0; 90.0] | 1,829 (92.3) [91.1; 93.5] | 1,908 (95.0) [94.1; 96.0] |
| **UK** |  |  |  |  |  |  |  |  |
| ***N prescriptions*** | ***2,367*** | **1,269** | ***945*** | ***530*** | ***2,007*** | ***1,026*** | ***735*** | ***403*** |
| Prescriptions with medication related to valproate indications | 1,572 (66.4) [64.5; 68.3] | 919 (72.4)  [70.0; 74.9] | 719 (76.1) [73.4; 78.8] | 419 (79.1) [75.6; 82.5] | 1,561 (77.8) [76.0; 79.6] | 854 (83.2)  [80.9; 85.5] | 654 (89.0) [86.7; 91.2] | 382 (94.8)  [92.6; 97.0] |
| ^†^ Prior medication within 12 months prior to valproate initiation  ^‡^ Prior medications related to epilepsy or bipolar disorder - within entire patient’s history prior to valproate initiation | | | | | | | | |

Table S2 Initiation of valproate as second line therapy in indication subgroups ‘epilepsy’ and ‘bipolar disorder’: proportion of valproate initiations preceded by other medications for valproate indication. Main study periods; all incident prescriptions and all first-ever prescriptions

|  | **Incident valproate prescriptions^†^** | | | | **First-ever valproate prescriptions^‡^** | | | |
| --- | --- | --- | --- | --- | --- | --- | --- | --- |
|  | **Epilepsy** | | **Bipolar disorder** | | **Epilepsy** | | **Bipolar disorder** | |
|  | **Main pre-implementation period n (%),  [95% CI of %]** | **Main post-implementation period n (%),  [95% CI of %]** | **Main pre-implementation period n (%),  [95% CI of %]** | **Main post-implementation period n (%),  [95% CI of %]** | **Main pre-implementation period n (%),  [95% CI of %]** | **Main post-implementation period n (%),  [95% CI of %]** | **Main pre-implementation period n (%),  [95% CI of %]** | **Main post-implementation period n (%),  [95% CI of %]** |
| **France** |  |  |  |  |  |  |  |  |
| ***N prescriptions*** | ***122*** | ***148*** | ***177*** | **163** | ***54*** | ***29*** | ***113*** | ***90*** |
| Prior medication related to valproate indication | 24 (19.7) [12.6; 26.7] | 11 (7.4) [3.2; 11.7] | 97 (54.8) [47.5; 62.1] | 74 (45.4) [37.8; 53.0] | 22 (40.7) [27.6; 53.8] | 12 (41.4) [23.5; 59.3] | 100 (88.5) [82.6; 94.4] | 75 (83.3) [75.6; 91.0] |
| **Germany PCP** |  |  |  |  |  |  |  |  |
| ***N prescriptions*** | ***446*** | ***557*** | ***150*** | ***199*** | ***306*** | ***353*** | ***113*** | ***139*** |
| Prior medication related to valproate indication | 94 (21.1) [17.4; 25.2] | 117 (21.0) [17.7; 24.6] | 58 (38.7) [30.8; 47.0] | 76 (38.2) [31.4; 45.3] | 77 (25.2) [20.4; 30.4] | 122 (34.6) [29.6; 39.8] | 68 (60.2) [50.5; 69.3] | 83 (59.7) [51.1; 67.9] |
| **Germany Neurologists/psychiatrists** |  |  |  |  |  |  |  |  |
| ***N prescriptions*** | ***553*** | ***577*** | ***259*** | ***266*** | ***237*** | **219** | ***184*** | ***170*** |
| Prior medication related to valproate indication | 146 (26.4) [22.8; 30.3] | 134 (23.2) [19.8; 26.9] | 165 (63.7) [57.5; 69.6] | 176 (66.2) [60.1; 71.8] | 116 (48.9) [42.4; 55.5] | 102 (46.6) [39.8, 53.4] | 164 (89.1) [83.7; 93.2] | 148 (87.1) [81.1; 91.7] |
| **Spain PCP** |  |  |  |  |  |  |  |  |
| ***N prescriptions*** | ***150*** | ***205*** | ***120*** | ***194*** | ***114*** | ***123*** | ***94*** | ***101*** |
| Prior medication related to valproate indications | 62 (41.3) [33.8; 49.3] | 97 (47.3) [40.6; 54.1] | 112 (93.3) [87.4; 96.6] | 176 (90.7) [85.8; 94.1] | 51 (44.7) [35.9; 53.9] | 76 (61.8) [53.0; 69.9] | 90 (95.7) [89.6; 98.3] | 100 (99.0) [94.6; 99.8] |
| **Spain Neurologists/psychiatrists** |  |  |  |  |  |  |  |  |
| ***N prescriptions*** | ***15*** | ***23*** | ***40*** | ***62*** | ***13*** | ***20*** | ***36*** | ***52*** |
| Prior medication related to valproate indication | 6 (40.0) [19.8; 64.3] | 16 (69.6) [49.1; 84.4] | 38 (95.0) [83.5; 98.6] | 53 (85.5) [74.7; 92.2] | 6 (46.2) [23.2; 70.9] | 16 (80.0) [58.4; 91.9] | 35 (97.2) [85.8; 99.5] | 51 (98.1) [89.9; 99.7] |
| **Sweden** |  |  |  |  |  |  |  |  |
| ***N prescriptions*** | ***1,461*** | ***1,521*** | ***2,370*** | ***2,764*** | ***1,188*** | ***1,193*** | ***1,969*** | ***2,098*** |
| Prior medication related to valproate indication | 689 (47.2) [44.6; 49.7] | 857 (56.3)  [53.8; 58.8] | 2,143 (90.4) [89.2; 91.6] | 2,535 (91.7) [90.7; 92.7] | 591 (49.7)  [46.9; 52.6] | 725 (60.8)  [58.0; 63.5] | 1,907 (96.9)  [96.1; 97.6] | 2,064 (98.4) [97.8; 98.9] |
| **UK** |  |  |  |  |  |  |  |  |
| ***N prescriptions*** | ***649*** | ***338*** | ***277*** | ***180*** | ***501*** | ***226*** | ***189*** | ***126*** |
| Prior medication related to valproate indications | 235 (36.2) [32.5; 39.9] | 149 (44.1) [38.8; 49.4] | 202 (72.9) [67.7; 78.2] | 140 (77.8) [71.6; 83.9] | 233 (46.5) [42.1; 50.9] | 123 (54.4)  [47.9; 61.0] | 172 (91.0) [86.9; 95.1] | 120 (95.2)  [91.5; 99.0] |
| ^†^ Prior medication related to epilepsy or bipolar disorder within 12 months prior to valproate initiation  ^‡^ Prior medications related to epilepsy or bipolar disorder - within entire patient’s history prior to valproate initiation | | | | | | | | |

**Table S3** Pregnancy counts in overall and exposed to valproate; entire 36-month pre- and post-implementation period

|  | **Entire pre-implementation period n (%)^†^** | **Entire post-implementation period**^‡^ **n (%)^†^** |
| --- | --- | --- |
| **France** |  |  |
| ***Pregnancies (total)*** | ***34*** | ***3*** |
| N pregnancies exposed to valproate | 20 (58.8) | 2 (66.7) |
| **Germany PCP** |  |  |
| ***N pregnancies (total)*** | ***1*** | ***0*** |
| N pregnancies exposed to valproate | 1 (100.0) |  |
| **Germany Neurologists/psychiatrists** |  |  |
| ***N pregnancies (total)*** | ***0*** | ***0*** |
| N pregnancies exposed to valproate |  |  |
| Spain PCP, Neurologists/psychiatrists |  |  |
| ***N pregnancies (total)*** | ***51*** | ***30*** |
| N pregnancies exposed to valproate | 37 (72.5) | 18 (60.0) |
| **Sweden**^‡^ |  |  |
| ***N pregnancies (total)*** | ***402*** | ***140*** |
| N pregnancies exposed to valproate | 179 (44.5) | 77 (55.0) |
| **UK** |  |  |
| ***N pregnancies (total)*** | ***435*** | ***177*** |
| N pregnancies exposed to valproate | 214 (49.2) | 85 (48.0) |
| **Overall** |  |  |
| ***N pregnancies (total)*** | ***923*** | ***350*** |
| N pregnancies exposed to valproate | 451 (48.9) | 182 (52.0) |
| ^†^ Percentage of total N pregnancies  ^‡^ Duration of the entire post-implementation period considered for analysis of pregnancy in Sweden was 23 months, | | |

**Appendix S4: Analysis of indication for valproate prescription**

Valproate indication was evaluated at prescription level.

A hierarchical proceeding was used to assign prescriptions to the indication categories.

Different approaches were used depending on whether prescriptions and diagnoses were linked or not linked in the data sources.

*France, Germany, Spain (diagnosis linked to prescription in the data sources)*

In the first step, only diagnoses linked to valproate prescription were considered for this analysis.

These diagnoses were assigned to two categories:

- 1. Indication of interest
- epilepsy
- bipolar disorder
- migraine headaches
  1. Other diagnoses associated with valproate prescription

If no diagnosis linked with the prescription was available, in the second step diagnosis records of relevant conditions (epilepsy, bipolar disorder, migraine headaches) were assessed within the entire available patient’s history in the data source +1 month after the valproate prescription date. In case of multiple relevant diagnoses, they were prioritised in the order as described above. If no diagnoses of relevant conditions were recorded within the above-mentioned time period, the indication for prescription was considered as “unknown”.

In summary, three categories were used:

1. Indication of interest
2. Other diagnosis
3. Indication unknown

A special case: ICD-10 codes which indicate a type of treatment or medical service use but not a diagnosis (e.g. ICD-10 code Z76.0 “Issue of repeat prescription”). In this case, the most recently recorded ICD-10 code indicating a diagnosis of interest, which was linked to valproate prescription was considered as indication for valproate.

If no diagnoses of interest linked to valproate prescriptions were identified in the history, the ICD-10 code Z76.0 “Issue of repeat prescription” was maintained and in the final step, all remaining prescriptions linked to the ICD-10 code Z76.0 were considered in the category “indication unknown”.

*UK and Sweden (diagnosis not linked to prescription in the data sources)*

In the first step diagnoses on the valproate prescription day were evaluated. If any diagnoses of interest was identified, this diagnosis was considered as the indication for valproate prescription.

In case multiple diagnoses were recorded on the prescription day the following hierarchy was used to assign a diagnosis to the prescription:

- Priority 1: epilepsy
- Priority 2: bipolar disorder
- Priority 3: migraine headaches

If no diagnoses of interest on the prescription day were identified, in the second step diagnosis records of relevant conditions (epilepsy, bipolar disorder, migraine headaches) were assessed within the entire available patient’s history in the data source +1 month after the valproate prescription date. In case of multiple relevant diagnoses, they were prioritised as described above. If no diagnoses of relevant conditions were recorded within the above-mentioned time period, the indication for prescription was considered as “unknown”.

In summary, two categories were used:

1. Indication of interest
2. Indication unknown

The indication category “other diagnoses” was not considered in the analysis for CPRD and Swedish registries data because no linkage between prescription and diagnosis is available in the data source.

**Appendix S5: List of Other Medications for Valproate Indications**

1. **Epilepsy**

| **Drug class** | **Substance** | **ATC WHO code** |
| --- | --- | --- |
| Seizure control | Carbamazepine | N03AF01 |
|  | Gabapentin | N03AX12 |
|  | Lacosamide | N03AX18 |
|  | Lamotrigine | N03AX09 |
|  | Levetiracetam | N03AX14 |
|  | Oxcarbazepine | N03AF02 |
|  | Phenobarbital | N03AA02 |
|  | Phenytoin | N03AB02  N03AB52 |
|  | Pregabalin | N03AX16 |
|  | Topiramate | N03AX11 |
|  | Zonisamide | N03AX15 |
|  | Clobazam | N05BA09 |
|  | Clonazepam | N03AE01 |
|  | Lorazepam | N05BA06  N05BA56 |
|  | Acetazolamide | S01EC01 |
|  | Bromide | M03AC06  M03AC08  M03AC09  N05CM11 |
|  | Eslicarbazepine acetate | N03AF04 |
|  | Ethosuximide | N03AD01  N03AD51 |
|  | Felbamate | N03AX10 |
|  | Fosphenytoin | N03AB05 |
|  | Mesuximide | N03AD03 |
|  | Perampanel | N03AX22 |
|  | Primidone | N03AA03 |
|  | Retigabine | N03AX21 |
|  | Rufinamide | N03AF03 |
|  | Stiripentol | N03AX17 |
|  | Sultiame | N03AX03 |
|  | Tiagabine | N03AG06 |
|  | Vigabatrin | N03AG04 |

1. **Bipolar Disorder**

| **Drug class** | **Substance** | **ATC WHO code** |
| --- | --- | --- |
| Antidepressants | Agomelatine | N06AX22 |
|  | Amitriptyline | N06AA09  N06CA01 |
|  | Bupropion | N06AX12  A08AA62 |
|  | Citalopram | N06AB04 |
|  | Clomipramine | N06AA04 |
|  | Doxepin | N06AA12 |
|  | Duloxetine | N06AX21 |
|  | Escitalopram | N06AB10 |
|  | Fluoxetine | N06AB03  N06CA03 |
|  | Imipramine | N06AA02 |
|  | Maprotiline | N06AA21 |
|  | Mianserin | N06AX03 |
|  | Mirtazapine | N06AX11 |
|  | Nortriptyline | N06AA10 |
|  | Paroxetine | N06AB05 |
|  | Reboxetine | N06AX18 |
|  | Sertraline | N06AB06 |
|  | Tranylcypromine | N06AF04 |
|  | Trimipramine | N06AA06 |
|  | Venlafaxine | N06AX16 |
| Mood stabilizers | Carbamazepine | N03AF01 |
|  | Lamotrigine | N03AX09 |
|  | Lithium | N05AN (N05AN01) |
| Typical neuroleptics | Haloperidol | N05AD01 |
| Atypical neuroleptics | Amisulpride | N05AL05 |
|  | Aripiprazole | N05AX12 |
|  | Asenapine | N05AH05 |
|  | Clozapine | N05AH02 |
|  | Olanzapine | N05AH03 |
|  | Paliperidone | N05AX13 |
|  | Quetiapine | N05AH04 |
|  | Risperidone | N05AX08 |
|  | Ziprasidone | N05AE04 |

1. **Migraine**

| **Drug class** | **Substance** | **ATC WHO code** |
| --- | --- | --- |
| Beta-blocker | Metoprolol | C07AB02  C07FB02  C07CB02  C07BB02  C07BB52  C07AB52 |
|  | Propranolol | C07AA05  C07FA05  C07BA05 |
|  | Bisoprolol | C07AB07  C07FB07  C07BB07  C07AB57  C09BX02 |
| Selective calcium-channel antagonist | Flunarizine | N07CA03 |
| Anti-epileptic | Topiramate | N03AX11 |
|  | Gabapentin | N03AX12 |
| Muscle relaxants | Onabotulinumtoxin A | M03AX01 (botulinum toxin) |
| Antidepressants | Amitriptyline | N06AA09  N06CA01 |
|  | Venlafaxine | N06AX16 |
| Nonsteroidal anti-inflammatory drugs | Naproxen | M01AE02  M01AE52  M01AE56 |
|  | Acetylsalicylic Acid | N02BA01  N02BA51  N02BA71 |
| Vitamins and electrolytes | Magnesium  vitamin B2 plus magnesium | A12CC  A11HA04 |
